# Prevalence of Blood Lead among Children Living in Battery Recycling Communities in Greater Jakarta, Indonesia

**DOI:** 10.3390/ijerph16071276

**Published:** 2019-04-10

**Authors:** Nurhayati A. Prihartono, Ratna Djuwita, Putri B. Mahmud, Budi Haryanto, Helda Helda, Tri Yunis Miko Wahyono, Timothy Dignam

**Affiliations:** 1 Department of Epidemiology, Faculty of Public Health, Universitas of Indonesia, Depok 16424, Indonesia; Djuwita257@gmail.com (R.D.); putrimachmud@gmail.com (P.B.M.); helda_dr@yahoo.com (H.H.); triyunis@yahoo.com (T.Y.M.W.); 2 Department of Environmental Health, Faculty of Public Health, Universitas of Indonesia, Depok 16424, Indonesia; bharyanto@ui.ac.id; 3 Division of Environmental Health Science and Practice, National Center for Environmental Health, U.S. Centers for Disease Control and Prevention, Atlanta, GA 30341, USA; ted9@cdc.com

**Keywords:** lead, battery recycling, environment, child, Jakarta, Indonesia

## Abstract

This study aimed to assess the prevalence of blood lead levels (BLLs) among children 1 to 5 years old who reside near and distant to informally used lead-acid battery (ULAB) recycling locations and examine risk factors for elevated BLLs. A cross-sectional study was conducted in three greater Jakarta neighborhoods where informal ULAB recycling occurs. Venous BLLs among 279 children were analyzed using portable blood lead testing machines. Demographic, child activities, and sources of lead exposure inside and outside homes were assessed. Multivariate analysis was performed to evaluate factors associated with the prevalence of BLLs. Forty-seven percent of children had BLLs ≥ 5 µg/dL and 9% had BLLs ≥ 10 µg/dL. No differences in geometric mean BLLs were observed between children who lived near and distant to ULAB locations. Older child age groups [Prevalence Ratio (PR) 2.14, 95% Confidence Interval (CI) 1.16, 4.18) and low household income (PR 1.58, 95% CI 1.03, 2.40) were associated with BLLs 5–9 µg/dL. Low educational attainment of the child’s father (PR 3.17, 95% CI 1.23, 8.16) and frequent outdoor child activity (PR 4.93, 95% CI 1.09, 22.21) were predictors of BLLs ≥ 10 µg/dL. This study shows the association between lead exposure among children and environmental sources. Public health officials can consider expanded surveillance, health care provider education, and development of strategies to reduce lead exposure.

## 1. Introduction

Lead is an environmentally persistent toxicant, which naturally exists in the environment; anthropogenic activities have increased the environmental burden and put populations at risk due to various sources of exposure. Exposure to lead can cause various nervous system symptoms, such as frequent headaches, nausea, colic, tremor, numbness of the limbs, and at high levels, lead lines on the gums [1,2,3]. Previous studies have correlated high blood lead levels (BLLs) with adverse effects on liver function, decreased kidney function, hypertension, and anemia among workers [4,5,6]. Intoxication from high levels of lead exposure has also caused death among children [7]. Children are more vulnerable to damage from lead exposure because they are growing [8]. In 2012, the U.S. Centers for Disease Control and Prevention (CDC) accepted their lead poisoning prevention advisory committee’s recommendation to replace the term “blood lead level of concern” with a reference BLL value of 5 μg/dL or higher [9].

Environmental sources of lead exposure include leaded gasoline and emissions from industries using lead as a raw material. During 2001–2006, to reduce lead exposure in the environment, Indonesia began phasing out the use of leaded gasoline. Over the past decade, the growth of car ownership has increased the demand for lead-acid batteries in Indonesia. Many of these batteries are recycled in the informal sector, leading to possible lead exposure among workers and children. 

Several studies that investigated BLLs among children living near used lead-acid battery (ULAB) recycling areas have found high BLLs in affected children [7,10,11]. Other sources of lead exposure include lead-based paint, lead-soldered cans, and water pipes, lead-glazed ceramics, and traditional medicines [12,13,14]. A 2018 study in Bangladesh among 385 young children (average age 28 months) reported 86% of children had BLLs above the CDC reference level of 5 μg/dL, and 35% of children were above 10 μg/dL [15]. A 2000 study also in Bangladesh among 779 students (range 4–12 years of age) reported a mean BLL of 15.0 µg/dL (range 4.2–63.1 µg/dL). Most students (87.4%) had BLLs ≥ 10 µg/dL [16]. A 2002–2003 study conducted in Bombay, India among 754 children under 12 years of age showed 33% had BLLs ≥ 10 μg/dL (geometric mean 8.4 μg/dL) [17]. Lastly, a 2012 study conducted in a pediatric hospital in Ho Chi Minh City, Vietnam hospital among 311 children aged <16 years reported a mean BLL of 4.97 μg/dL and 7% of the participants having BLLs greater than 10 μg/dL [18].

Mean BLLs among children living in Jakarta are expected to be higher than in similar countries because the lead in gasoline was not phased out in Indonesia until 2006 [19]. A 1996 study conducted among 161 elementary school age Jakarta children (average age 7 years) reported that 27% of children had BLLs ≥ 10 µg/dL; mean BLL was 7.7 μg/dL [20]. A 2002 study conducted among second- and third-grade school children in Jakarta, Indonesia, found an average BLL of 8.6 μg/dL [21]. Also, the presence of ULAB recycling areas is potentially contributing to higher mean child BLLs in greater Jakarta. A 2015 report estimated that 71 battery-recycling smelters exist in greater Jakarta [16]. ULAB recycling mostly occurs in the informal sector. Such recycling activities often take place very close to neighborhoods, with minimal control from the government. To date, Indonesia does not have a child BLL surveillance system. Limited data are available on BLLs throughout Jakarta and among children living near informal sector smelters. This is the first study in Indonesia investigating BLLs among children ages 1–5 years through house-to-house data collection and blood lead measurement. 

The objectives of this study are to:

(1) Obtain a prevalence estimate of BLLs among children ages 1–5 years living in communities affected by ULAB recycling;
(2) Identify risk factors and sources of lead exposure in these children.

## 2. Materials and Methods 

We conducted a cross-sectional study in Greater Jakarta, Indonesia, during 2–10 April 2015. The study included blood lead sampling, visual observation, and a questionnaire to gather information on the demographics and risk factors for lead exposure. The University of Indonesia (No. 130/H2.F10/PPM.00.02/2015, 9 March 2015) and CDC institutional review boards (Approval Protocol No. 6344, 13 December 2013) approved the study protocol.

### 2.1. Enrollment of Children/Participant Inclusion Criteria

The study population included children ages 1–5 years who were living in three neighborhoods (subdistricts of Pegangsaan Dua, Cipondoh, and Dadap) where ULAB recycling occurs. Each neighborhood had a known ULAB recycling site. We obtained a roster of children aged 1–5 years from the local health service post (Posyandu cadre) to randomly select children. Of 233 eligible children who lived <200 m (i.e., near) from an informal ULAB recycling site, 187 were invited to participate in the study. An additional 174 out of 200 children who lived 200–250 m (i.e., distant) from informal ULAB recycling sites also were invited to be in the study. We had a refusal rate of 22.1%. Fathers of 42 children who lived near ULAB recycling sites and 30 who lived distant from ULAB recycling sites refused to participate. The fathers of eight different children did not want blood drawn. Occupation and other potential lead exposure risk factors were not collected among families who refused to participate. In addition, two children moved away before they could participate. In all, 279 children (92 in Pegangsaan Dua, 92 in Cipondoh, and 95 in Dadap) were enrolled and included in the analysis. 

### 2.2. Blood Lead Collection and Follow-Up

Each of two study teams included a phlebotomist, an interviewer, and an environmental inspector and sampler. Study teams went house-to-house to collect data from the roster of randomly selected households. A phlebotomist conducted venous blood sampling for this study. For the procedure, each child’s arm was washed with soap and water, dried with a paper towel, wiped with an alcohol prep swab, and dried with gauze. Paper towel packaging was labeled as “chemical free.” The vein was punctured with a sterile, non-reusable needle. About 3 mL of blood was collected in a tube for lead analysis. After obtaining the sample, the phlebotomist applied an adhesive bandage to the puncture site. Field teams used a LeadCare II © portable blood lead testing machine (Magellan Diagnostics, Cambridge, MA) to analyze blood lead samples. To avoid potential contamination during transport, blood samples were analyzed inside the home. For quality assurance, venous blood samples were split for approximately 15% (*n* = 42) of randomly selected children in the study. Split venous samples were analyzed both on LeadCare II © portable blood lead testing machines and by the Prodia Occupational Health Institute using inductively coupled plasma mass spectrometry. Split venous sample results were correlated (R = 0.82). Children with BLLs ≥ 5 µg/dL were referred to the Health Care Unit for medical consultation and follow-up blood lead testing. Parents were provided with nutritional and environmental education. Cookies with soya powder were given to parents for their children’s additional nutrition.

### 2.3. Variable Descriptions and Data Analysis

We used descriptive statistics to examine categories of child BLLs (<5 μg/dL, 5–9 μg/dL, and ≥10 μg/dL) and geometric mean BLLs to assess distance to ULAB recycling sites. Blood lead levels were non-normally distributed. We examined BLL categories of children by age, sex, family income status, mother and father’s education level (low if junior high school or less and high if senior high school or above), mother and father’s occupation, children’s activities indoors and outdoors, sources of lead exposure, and distance to the nearest ULAB recycling site. We used the chi-squared test statistic to compare proportions of BLLs categories of some variables. We asked about the type of dishware used in the home (e.g., ceramic, earthenware, plastic, steel, aluminum, or glass). Based on prior unpublished assessments, glass dishware was categorized as potentially lead-containing. A swab test was not used to assess lead contamination of dishware. We considered sources of drinking and cooking water, which we categorized as improved and unimproved. Improved drinking water sources included public taps or standpipes, tube wells or boreholes, protected dug wells, and protected spring and rainwater collection sites. The unimproved drinking water sources included unprotected dug wells, unprotected spring carts with small tanks or drums, tanker trucks, surface water (e.g., rivers, dams, lakes, canals, streams, and irrigation channels), and refilled bottled water (the quality of which is difficult to assess) [22]. Bottled water used for drinking and cooking is obtained from refill centers. Quality of drinking water (good, poor) was assessed through visual observation and source information. We also assessed the water delivery method to the household. The categories were polyvinyl chloride, metal, and plastic pipes, and refilled bottle water. We performed a multivariate analysis, a modification of Cox proportional hazard, by assigning a constant risk period [23], to identify predictors of BLLs 5–9 μg/dL and ≥10 μg/dL. We began the analysis by including all independent variables in the model, regardless of the association in bivariate analysis. We treated the children’s outside activity variable as an ordinal scale. We assessed interactions between variables. We considered variables that significantly predicted child BLLs (*p* < 0.05) for inclusion in the final model.

## 3. Results

We found that 47% of study children had BLLs ≥ 5 µg/dL and 9% of those children had BLLs ≥ 10 µg/dL. There was no difference in the proportion of children with BLLs ≥ 10 µg/dL compared to children with BLLs < 5 µg/dL by study neighborhood (*p* = 0.41). There was a significant difference in the proportion of children with BLLs 5–9 µg/dL compared to children with BLLs < 5 µg/dL by study neighborhood (*p* = 0.01) (Table 1). However, the study neighborhood was not significant in multivariate modeling. Children who lived far from and near ULAB recycling sites had similar geometric mean BLLs (4.90 µg/dL and 4.69 µg/dL, respectively, *p* = 0.63). 

Male (54%) and older children (62% of children aged 5 years) had the highest prevalence of BLLs ≥ 5 µg/dL (Table 2). Children whose father had junior high school or less education level had a higher proportion of BLLs ≥ 5 µg/dL (54%) than did children whose father was highly educated (41%) (*p* = 0.038).

Eighty-five percent of mothers served as homemakers and only 15% had jobs. The proportion of children with BLLs 5 µg /dL or greater was lower among children with working mothers (34%) than children with homemaker mothers (50%) (*p* = 0.068). Based upon job status of the fathers, children with unemployed fathers had a higher percentage of BLLs 5 µg /dL or greater (60%) compared to children with working fathers (47%) (*p* = 0.566). 

Our study results show that 65% of children in the lowest household income category [Rupiah (Rp) 1,500,000 or less, or about $110 per month] had BLLs ≥ 5 µg/dL. Children in families with a monthly household income greater than Rp 3,500,000 (about $260/month) had the lowest proportion of BLLs ≥ 5 µg/dL (*p* = 0.004).

The proportion of children with BLLs ≥ 5 µg/dL were similar among those in households in which a member had a lead-related job (41%) or hobby (47%), and in households using dishes that potentially contained lead (45%) (Table 3). Among children living near ULAB recycling sites, 16% had BLLs ≥ 5 µg/dL. Among children whose homes were cleaned daily, 47% had BLLs ≥ 5 µg/dL. In contrast, 33% of children whose homes were cleaned weekly had BLLs ≥ 5 µg/dL. Compared with children living in homes that were renovated (inside or outside the home during the last 6 months), children who did not have their home renovated had a higher proportion of BLLs ≥ 5 µg/dL (55% vs. 46%) (*p* = 0.294). We found no association between BLLs and number of electronic appliances owned.

More children (range 72–85%) lived in homes with non-intact paint across the three location categories where paint quality was assessed (Table 3). Among children living in homes that had cracked window paint, 46% had BLLs ≥ 5 µg/dL. Fifty-one percent of children in homes with intact window paint had BLLs ≥ 5 µg/dL. We also saw a similar pattern of BLLs ≥ 5 µg/dL when comparing children whose homes had cracked indoor paint to those in homes with intact indoor paint. Conversely, 49% of the children whose homes had cracked outdoor paint compared with 39% of children in homes with intact outdoor paint had BLLs ≥ 5 µg/dL.

Among children who had a parent observe their child eating/mouthing non-food items, 50% had BLLs ≥ 5 µg/dL (Table 3). As the duration of outdoor activity increased, so did the percentage of children with BLLs ≥ 10 μg/dL. The percentages of children with BLLs < 10 μg/dL did not reveal a correlating pattern with duration of outdoor activity.

Children who had unimproved drinking water supplies had a higher proportion of BLLs ≥ 5 μg/dL than those who had improved drinking water available (51% vs. 35%) (*p* = 0.020). Children who used poor quality drinking water had a higher proportion of BLLs ≥ 5 μg/dL than those who had good quality drinking water available (61% vs. 46%) (*p* = 0.134). Drinking water delivery type was similar among households with children who had elevated BLLs. Children with BLLs ≥ 5 μg/dL had polyvinyl chloride pipes (61/120, 51%), plastic pipes (15/31, 48%), and bottled water (54/117, 46%) as their primary drinking water delivery type. A metal pipe water service delivery was less common in households among children with BLLs ≥ 5 μg/dL (2/11, 18%) (Table 4). 

Fathers educated to junior high school or less and households reporting a longer duration of their child’s outdoor activity best predicted child BLLs ≥ 10 µg/dL (*p* = 0.02 and 0.04, respectively). In addition, increasing age, low household income, and unimproved drinking water supply were factors predicting BLLs of 5 to 9 µg/dL (*p* = 0.01, 0.03, and 0.05, respectively) (Table 5).

## 4. Discussion

This cross-sectional, home-based study is the first in Jakarta to examine blood lead levels among very young children. This study shows that 47% of children who live in communities potentially affected by ULAB recycling have BLLs for which public health action should begin, as recommended by the CDC. Children at 5 years of age (compared to children at 1 year of age), low household income, low father educational attainment, and children spending more time in outdoor activities were predictors of elevated BLLs among children. Unimproved drinking and cooking water sources were also associated with child elevated BLLs but were not statistically significant.

The prevalence of BLLs ≥ 5 μg/dL among young children from greater Jakarta in this study was higher than that of U.S. children ages 1–5 years (47% vs. 1.3%, CDC, unpublished data). However, the prevalence of BLLs ≥ 10 μg/dL among children was lower than among persons who lived near battery recycling sites in Senegal and Vietnam [7,10]. A 2007–2008 study among 50 children and 31 adults in a community involved in the recycling of ULAB in the suburbs of Dakar, Senegal reported a very high mean BLL (129.5 µg/dL) [4]. A 2015 study in Vietnam included children up to 10 years old and reported that 28% of the children had BLLs ≥ 45 μg/dL [10].

We observed that children in households that used an unimproved water source for drinking and cooking had an increased risk of having BLLs 5–9 µg/dL. For this study, we defined an unimproved water source as a well without cement walls, into which potentially lead-contaminated soil could enter the water supply. An unimproved water source can also be a source of contamination for infectious and non-infectious diseases. A study in Bangladesh found high arsenic concentrations in unprotected wells [24]. In this study, we found a similar proportion of BLLs ≥ 5 μg/dL among children based on water delivery method. Thus, potential lead exposure could be due to water that came from lead-contaminated wells, where the purification process was not known. Additionally, in a previous study in Jakarta among second- and third-grade children, Albalak et al. (2003) found an association between children with BLLs ≥ 10 µg/dL and drinking water source [21]. 

We found that child BLLs increased with child age. Children who were age 5 years had the highest proportion (62%) of BLLs 5–9 µg/dL. One-year-old children had the highest proportion (65%) of BLLs < 5 µg/dL. This finding is consistent with a 2017 study in a community with lead smelting, battery production and production of lead-containing chemicals in China that found the highest BLLs were among children ages 3–5 years [25]. The increase in BLLs with age might result from older children being more likely to have activities outside the home, creating greater contact with potential sources of lead exposure (e.g., soil and dust). A 2012 study in an area in southern Brazil with high concentrations of lead in soil among 97 children ages 1–5 years, reported higher BLLs among older children and hypothesized that the cause was due to increased outdoor activity among older children [26].

We found that the longer a child was reported to be active outside the home, the higher their BLL. Outdoor activities are an integral part of a child’s learning experience. During normal activities, children exhibit a wide variety of behaviors (e.g., hand-in-mouth or object-in-mouth), which might increase the risk for lead contamination through ingestion of non-food objects. Ko and colleagues (2007) found a positive correlation between children’s hand-to-mouth and object-to-mouth behaviors and their BLL [27]. However, our study did not find a significant association of ingestion of non-food items with the BLLs of children in multivariate models. Air lead from ULABs is another potential exposure pathway.

This study is consistent with another study that found lower educational level attainment of the father was associated with higher BLLs of children [28]. We observed children whose father had an education level of junior high school or less were three times more likely to have BLLs ≥ 10 μg/dL than were children of a higher educated father (prevalence ratio = 3.17, 95% confidence interval = 1.23 to 8.16). In addition, we found an increase of BLLs among children as household income decreased. Other studies have also shown an association between poverty and elevated BLLs among children [28,29]. Our study was conducted in three poor and crowded neighborhoods, with relatively homogenous socioeconomic backgrounds. However, socioeconomic factors (i.e., household income and father’s education level) were independently associated with elevated BLLs among children. Several factors are thought to be correlated with socioeconomic disadvantages among children with elevated BLLs. These factors include low awareness of environmental hazards, children’s personal hygiene, and nutritional deprivation (e.g., low calcium intake and iron deficiency) [30,31].

Limitations of this study are that we did not measure air lead levels in the surrounding environment. Therefore, we could not assess the contribution of potential air lead levels to lead in the home environment from the informal lead battery recycling processes. This study, however, did collect potential lead exposure risk factors including visual observation of interior and exterior paint, characteristics of drinking and cooking water, distance to ULAB recycling centers, household occupations and hobbies, and demographic factors. In Jakarta, leaded gasoline was phased out during July 2006. We hypothesize that leaded gasoline has contaminated soil and water over time. It is unclear if lead-based paint may have been used to paint homes in Jakarta. Results from a 2019 study conducted in 13 greater Jakarta neighborhoods from 103 homes and 19 preschools showed only 2.7% of X-ray fluorescence paint measurements and 0.05% of dust wipe samples exceeded the commonly applied U.S. Environment Protection Agency guideline values for paint and dust [32]. The present study failed to detect any association between cracked interior or exterior paint (through visual observation) with children’s BLLs. This study would have been strengthened if the measurement of lead in paint, water, and soil had been assessed. A final study limitation is the fact that LeadCare II portable instruments were used to analyze venous blood samples. Magellan Diagnostics recalled LeadCare II Testing Systems because they may underestimate venous blood samples and give falsely lower test results (https://www.fda.gov/MedicalDevices/Safety/ListofRecalls/ucm561936.htm). Therefore, we may have reported lower BLLs in the study population.

## 5. Conclusions

This study contributes information about BLLs 5–9 and ≥10 µg/dL among children ages 1–5 years who live around informal lead recycling activity sites in greater Jakarta. For child BLLs 5–9 µg/dL, older child age and lower household income were the main predictors. Lower father education level and frequent outside child activity were the main predictors of child BLLs ≥ 10 µg/dL. To identify and control lead sources the following should be considered: blood lead testing and surveillance of children in greater Jakarta, governmental and parental attention, pediatric health care provider education about the importance of testing children for blood lead, testing water for lead content and inspection of homes of children with BLLs of 5 μg/dL or more.

## Figures and Tables

**Table 1:** Distribution of blood lead levels among children ages 1–5 years in three neighborhoods of Jakarta, Indonesia, 2015 (*n* = 279)**.**

			Neighborhood						
Blood Lead Level Category (µg/dL)	Total		Cipondoh		Dadap		Pegangsaan Dua		
	n	%	n	%	n	%	n	%	*p*-Value ^a^
<5	147	52.7	59	64.1	41	43.2	47	51.0	ref
5–9	106	38.0	26	28.3	46	48.4	34	37.0	0.01
≥10	26	9.3	7	7.6	8	8.4	11	12.0	0.41

^a^ chi-square test, *p* value < 0.05 is defined as significant.

**Table 2:** Percentage of blood lead levels among children ages 1–5 years by demographic characteristic —Jakarta, Indonesia, 2015 (*n* = 279)**.**

Variable	Blood Lead Level Category (µg/dL)							
	<5		5–9		≥10		Total	
	n	% ^b^	n	%	n	%	n	%
**Age (years)**								
1	41	65	16	25	6	10	63	23
2	36	52	28	41	5	7	69	25
3	27	53	17	33	7	14	51	18
4	27	50	22	41	5	9	54	19
5	16	38	23	55	3	7	42	15
**Sex**								
Male	74	46	68	42	20	12	162	58
Female	73	62	38	33	6	5	117	42
**Mother’s Education**								
Junior high school or less	87	49	70	40	19	11	176	63
Senior high school or above	60	58	36	35	7	7	103	37
**Father’s Education**								
Junior high school or less	63	46	53	39	20	15	136	49
Senior high school or above	84	59	53	37	6	4	143	51
**Mother’s occupation**								
None	120	50	95	40	23	10	238	85
Office worker, trader, police/military	12	67	4	22	2	11	18	7
Labor, farmer, fisherman	6	60	4	40	0	0	10	3
Other	9	69	3	23	1	8	13	5
**Father’s Occupation**								
None	2	40	2	40	1	20	5	2
Office worker, trader, police/military	48	55	31	35	9	10	88	32
Labor, farmer, fisherman	35	51	23	34	10	15	68	25
Other	62	53	50	42	6	5	118	41
**Household Income**								
≤1,500,000	24	35	31	46	13	19	68	25
>1,500,000–≤2,500,000	64	57	44	39	4	4	112	40
>2,500,000–≤3,500,000	33	57	19	33	6	10	58	21
>3,500,000	26	64	12	29	3	7	41	14
**Distant to ULAB recycling area (meters)**								
Near (<200)	75	54	53	39	10	7	138	49
Distant (200–250)	72	51	53	38	16	11	141	51

^b^ Percentages are rounded; ULAB=Used Lead Acid Battery.

**Table 3:** Prevalence of blood lead level categories among children ages 1–5 years by potential sources of lead exposure and children’s activity—Jakarta, Indonesia, 2015 (*n* = 279)**.**

	Blood Lead Level Category (µg/dL)							
Variable	<5		5–9		≥10		Total	
	n	% ^b^	n	%	n	%	n	%
**Lead-Related Work**								
No	110	51	85	39	21	10	216	77
Yes	37	59	21	33	5	8	63	23
**Lead-Related Hobby**								
No	22	54	16	39	3	7	41	15
Yes	125	52	90	38	23	10	238	85
**Dishware Potentially Contains Lead**								
No	44	54	28	34	10	12	82	29
Yes	103	52	73	40	16	8	197	71
**House Cleaning**								
Daily	145	53	105	38	26	9	276	99
Weekly	2	67	1	33	0	0	3	1
**Home Renovation During Previous Six Months**								
Yes	19	45	20	48	3	7	42	15
No	128	54	86	36	23	10	237	85
**Electronics**								
0					1	100	1	< 1
1–2	55	47	50	42	13	11	118	42
3–4	87	59	49	33	11	8	147	53
5–6	5	38	7	54	1	8	13	5
**Window Paint**								
Intact	38	49	34	43	6	8	78	28
Cracked	109	54	72	36	20	10	201	72
**Indoor Paint**								
Intact	24	45	14	48	4	7	42	15
Cracked	123	52	92	39	22	9	237	85
**Outdoor Paint**								
Intact	30	61	14	29	5	10	49	18
Cracked	117	51	92	40	21	9	230	82
**Eating/mouthing Non-Food Items**								
No	87	55	62	39	10	6	159	57
Yes	60	50	44	37	16	13	120	43
**Children Outside Activity (hours)**								
0–<2	46	61	26	35	3	4	75	27
2–<5	68	52	53	40	10	8	131	47
5–<7	27	54	14	28	9	18	50	18
≥7	6	26	13	57	4	17	23	8

^b^ Percentages are rounded.

**Table 4:** Prevalence of blood lead level (BLL) categories among children ages 1–5 years, by drinking water characteristics—Jakarta, Indonesia, 2015 (*n* = 279).

Drinking Water Characteristic	Blood Lead Level Category (µg/dL)						Total	
	<5		5–9		≥10			
	n	% ^b^	n	%	n	%	n	%
**Drinking Water Supply**								
Improved	43	65	17	26	6	9	66	24
Unimproved	104	49	89	42	20	9	213	76
**Quality of Drinking Water**								
Good quality	136	54	90	36	25	10	251	90
Poor quality	11	39	16	57	1	4	28	10
**Water Delivery Type Supplying Drinking Water**								
PVC pipe	59	49	46	38	15	13	120	43
Metal pipe	9	82	2	18	0	0	11	4
Plastic pipe	16	51	12	39	3	10	31	11
Refilled water bottle	63	54	46	39	8	7	117	42

^b^ Percentages are rounded, PVC= Polyvinyl Chloride.

**Table 5:** Final multivariate model of predictors of blood lead levels (BLLs) of 5–9 µg/dL and ≥ 10 µg/dL, relative to BLLs < 5 µg/dL, among children in Jakarta, Indonesia, 2015 (*n* = 279).

Blood Lead Level 5–9 µg/dL		
Variable	Prevalence Ratio (95% CI)	*p* Value
**Age (Years)**		
1	1.00	
2–4	1.59 (0.92–2.74)	0.09
5	2.14 (1.16–4.18)	0.01
**Household income**		
>Rp 1,500,000	1.00	
≤Rp 1,500,000	1.58 (1.03–2.40)	0.03
**Water supply**		
Improved	1.00	
Unimproved	1.68 (1.00–2.82)	0.05
**Blood Lead Level ≥ 10 µg/dl**		
**Father’s education level**		
Senior high school or above	1.00	
Junior high school or less	3.17 (1.23–8.16)	0.02
**Children’s outside** **activity (hours)**		
0–<2	1.00	
2–<5	2.43 (0.66–8.86)	0.18
5–<7	3.53 (0.92–13.08)	0.06
≥7	4.93 (1.09–22.21)	0.04

CI = Confidence Interval, *p* value < 0.05 is defined as significant
